# Modeling Pan Evaporation for Kuwait by Multiple Linear Regression

**DOI:** 10.1100/2012/574742

**Published:** 2012-11-22

**Authors:** Jaber Almedeij

**Affiliations:** Civil Engineering Department, Kuwait University, P.O. Box 5969, 13060 Safat, Kuwait

## Abstract

Evaporation is an important parameter for many projects related to hydrology and water resources systems. This paper constitutes the first study conducted in Kuwait to obtain empirical relations for the estimation of daily and monthly pan evaporation as functions of available meteorological data of temperature, relative humidity, and wind speed. The data used here for the modeling are daily measurements of substantial continuity coverage, within a period of 17 years between January 1993 and December 2009, which can be considered representative of the desert climate of the urban zone of the country. Multiple linear regression technique is used with a procedure of variable selection for fitting the best model forms. The correlations of evaporation with temperature and relative humidity are also transformed in order to linearize the existing curvilinear patterns of the data by using power and exponential functions, respectively. The evaporation models suggested with the best variable combinations were shown to produce results that are in a reasonable agreement with observation values.

## 1. Introduction

Estimation of the water loss by evaporation is important for modeling, survey, and management of many projects related to hydrology and water resources systems [1, 2]. For example, in many arid and semiarid countries in the Middle East, domestic and industrial water are mostly obtained by desalination. This is an expensive process and other water sources such as harvested rainwater and treated wastewater are being considered for landscape irrigation and other purposes. These projects, however, may require large volumes of water to be stored in artificial lakes and reservoirs open to the atmosphere. In the arid regions with flat terrains, suitable reservoir sites will be limited, and the reservoirs may be shallow with large surface areas. In these cases, significant amounts of water may be lost due to evaporation [3, 4]. Consequently, determination of evaporation losses will be critical in the assessment of the design and operation of these reservoirs.

Evaporation depends on the supply of heat energy and vapor pressure gradient, which in turn depend on meteorological factors such as temperature, relative humidity, wind speed, atmospheric pressure, and solar radiation [5]. In hydrological practice, actual evaporation may be measured by using pan evaporimeters. However, in arid areas of sufficiently low humidity, it is widely acknowledged the existence of a complementary relationship that can influence the reliability of the measurements of pan evaporation [6, 7]. When water availability is limited, actual (regional) evaporation falls below its potential, and a certain amount of energy becomes available. This energy excess increases the temperature and humidity gradients of the overpassing air and leads to an increase in potential evaporation equal in magnitude to the decrease in actual evaporation. If water availability is increased, actual evaporation increases while potential evaporation decreases. This process continues until the actual and potential evaporations become equivalent upon the prevailing conditions of moisture availability. The latter condition is likely present in arid coastal countries where moisture availability is high enough by which pan evaporation measurements become nearly representative to actual evaporations.

Models to estimate evaporation based on meteorological data have also been used by many researchers [8–10]. The well-known Penman's equation may be recommended as a standard method [11]. Penman derived constants for water bodies based on research in lakes. However, to solve this equation, information is needed not only on the external meteorological conditions, but also on the heat storage within the water body, which requires temperature profile measurements within the water [7, 12]. Accordingly, Penman equation cannot be applied if one or more of its parameters are not available from meteorological weather station measurements. 

For this reason, several simpler analytical and empirical equations have been developed [2, 5, 13–17]. For example, Cahoon et al. [14] and Fennessey and Vogel [18] employed regression methods to develop models for regional monthly average evaporation in USA as a function of readily available variables such as temperature and site's longitude and elevation. The empirical models were shown to be an improvement over other temperature-based models such as that of Linacre [19] and that of Hargreaves and Samani [20]. Tabari et al. [17] estimated evaporation in semiarid region of Iran using both techniques of artificial neural network and of multivariate nonlinear regression. The techniques comprise various combinations of meteorological variables of temperature, relative humidity, wind speed, solar radiation, and precipitation. They showed that both techniques provide acceptable results. Other similar attempts were also made successfully in the arid region of Saudi Arabia [21–23].

 The aim here is to develop simple empirical relations for the estimation of daily and monthly evaporation in Kuwait in terms of available meteorological data. Pan evaporation will be used as the dependent variable. Multiple linear regression techniques will be employed to fit the possible model forms. The performance of the models chosen with the best independent variable combinations will then be compared and discussed.

## 2. Meteorological Data

Kuwait, which is about 18,000 km^2^, is a desert country characterized by long, hot, and dry summers and short winters. The average depth of annual evaporation is high approaching a value of 4000 mm, while that of precipitation is low varying from 50 mm to 250 mm. Temperature during summer (winter) reaches an average daily high of 43°C (15°C), with the average daily low falling to 23°C (5°C). Winter temperatures can be classified as mild, but occasionally become cold when northerly or north-westerly winds bring cold air from the north. Summers are uniformly hot, and temperatures can be very high when hot winds blow from the desert. Owing to the coastal location of the country, the heat is often rendered even more uncomfortable by high humidity approaching a maximum of 90 percent or more.

**Figure 1:**
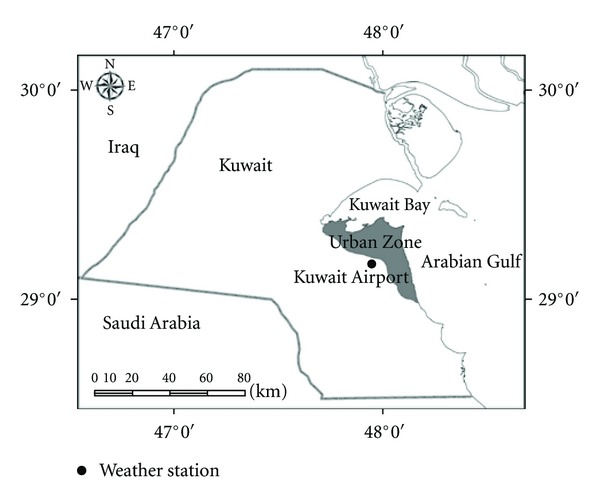
Location of the weather station in Kuwait.

The meteorological data used in this study are daily average measurements of pan evaporation (mm day^−1^), temperature at 2 m height (°C), relative humidity (%), and wind speed at 2 m height (m s^−1^). The effect of precipitation on evaporation rates will not be considered in this study due to the rare rainfall events in the country [24]. The climatological data adopted are readily available from the Meteorological Department of the Directorate of Civil Aviation collected at a local weather station near Kuwait Airport (Figure 1). These data are of substantial continuity coverage, within a period of 17 years between January 1993 and December 2009 and considered representative of the climate within the urban zone. The reason for such a meteorological point estimate to be considered representative is that the urban zone of the country spans a small area, within latitudes from 29°20′N to 29°03′N and longitudes from 47°37′E to 48°10′E, and is characterized by nearly flat surface elevations. It should also be mentioned that the data for relative humidity provided by this weather station includes only the daily maximum and minimum measurements. Accordingly, the average daily values considered here are obtained by calculating the arithmetic mean of the maximum and minimum measurements.  Table 1 presents statistical description for the daily average measurements of the climatological parameters. It can be seen that within the specified time duration, the measurements for each parameter are highly variable reflecting the typical arid climate of the country.

The relation between evaporation and the climatological parameters of temperature, relative humidity, and wind speed can be described by considering a correlation analysis.  Table 2 shows that the temperature has the highest correlation coefficient with evaporation of +0.86. This is not a surprising result for a desert country since the physical mechanisms responsible for evaporation are directly proportional to the heat provided by high temperature conditions. The relative humidity has the second highest correlation coefficient with evaporation equaling −0.81. This high correlation reflects the coastal location of the country along the Arabian Gulf. Relative humidity refers to the amount of water in the air, as a fraction of the total amount a saturated air can hold. The more humidity is in the air, the less space will be available for evaporation. Once the air reaches an upper limit of 100 percent relative humidity, it is no longer able to hold additional water molecules. The wind speed has the lowest correlation coefficient with evaporation of +0.53. The speed at which wind flows over the water surface affects the rate of evaporation by sweeping away water particles that are in the air, allowing more particles to evaporate in the space above the water surface.

## 3. General Model Form

Multiple linear regression techniques can be used to model the pan evaporation data for Kuwait in terms of the local climatological parameters of temperature, relative humidity, and wind speed. For a multiple linear regression model, the dependent variable *y* is assumed to be a function of *k* independent variables *x*_1_, *x*_2_, *x*_3_,…, *x*_*k*_. The model is expressed in the form

(1) $$\begin{array}{c} y_{i}=b_{0}+b_{1}x_{1,i}+\cdots +b_{k}x_{k,i}+e_{i}, \end{array}$$

where *b*_0_, *b*_1_,…, and *b*_*k*_ are fitting constants; *y*_*i*_, *x*_1,*i*_,…, *x*_*k*,*i*_ represent the *i*th observations of each of the variables *y*, *x*_1_,…, *x*_*k*_, respectively; *e*_*i*_ is a random error term representing the remaining effects on *y* of variables not explicitly included in the model. For simple regression models, *e*_*i*_ can be assumed to be an uncorrelated variable with zero mean. The most common procedure for estimating the values of *b*_0_, *b*_1_,…, and *b*_*k*_ is to employ the least squares criterion with the minimum sum of squares of error terms (S); that is to find *b*_0_, *b*_1_,…, and *b*_*k*_ to minimize

(2) S=∑i=1n(yiobserved−b0−b1x1,i−⋯−bkxk,i)2=∑i=1n(yiobserved−yicalculated)2=∑i=1nei2.

As a result, *b*_0_, *b*_1_,…, and *b*_*k*_ must satisfys

(3) $$\begin{array}{c} \frac{\partial S}{\partial b_{j}}=2\sum_{i=1}^{n}e_{i}\frac{\partial e_{i}}{\partial b_{j}}=0,\;j=0,1,\ldots ,k \end{array}$$

and since *e*_*i*_ = *y*_*i*__observed_ − *y*_*i*__calculated_, the above equation becomes

(4) $$\begin{array}{c} \frac{\partial S}{\partial b_{j}}=-2\sum_{i=1}^{n}e_{i}\frac{\partial {y_{i}}_{\text{calculated}}}{\partial b_{j}}=0,\;j=0,1,\ldots ,k. \end{array}$$

**Figure 2:**
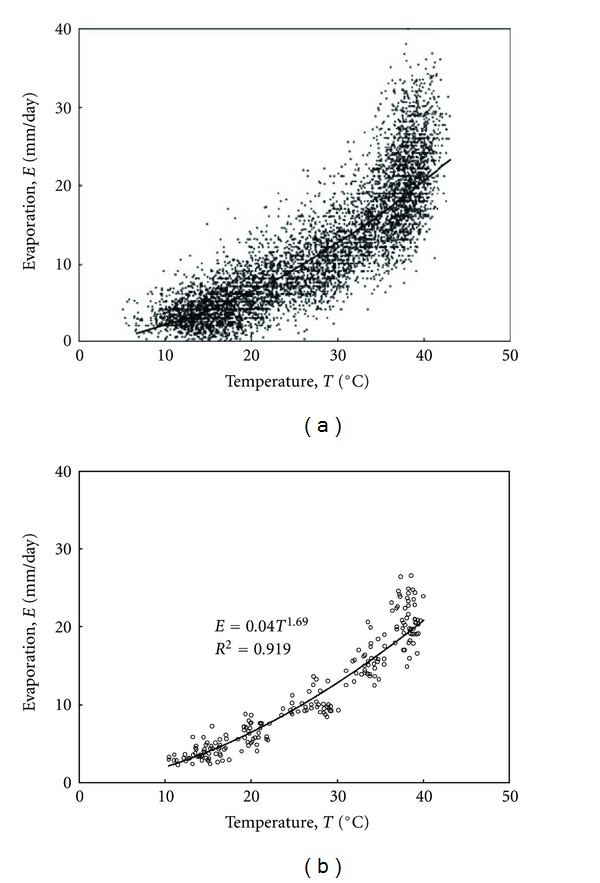
Evaporation as a function of temperature for the data of Kuwait from 1993 to 2009. The solid curves represent suggested relations fitted using regression analysis. (a) Daily average data; (b) monthly average data.

**Figure 3:**
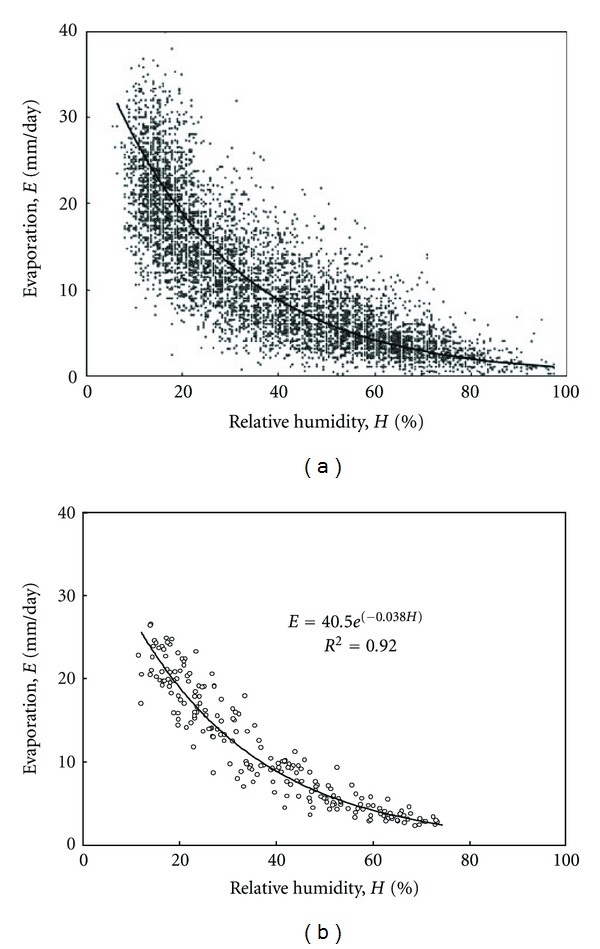
Evaporation as a function of relative humidity for the data of Kuwait from 1993 to 2009. The solid curves represent suggested relations fitted using regression analysis. (a) Daily average data; (b) monthly average data.

**Figure 4:**
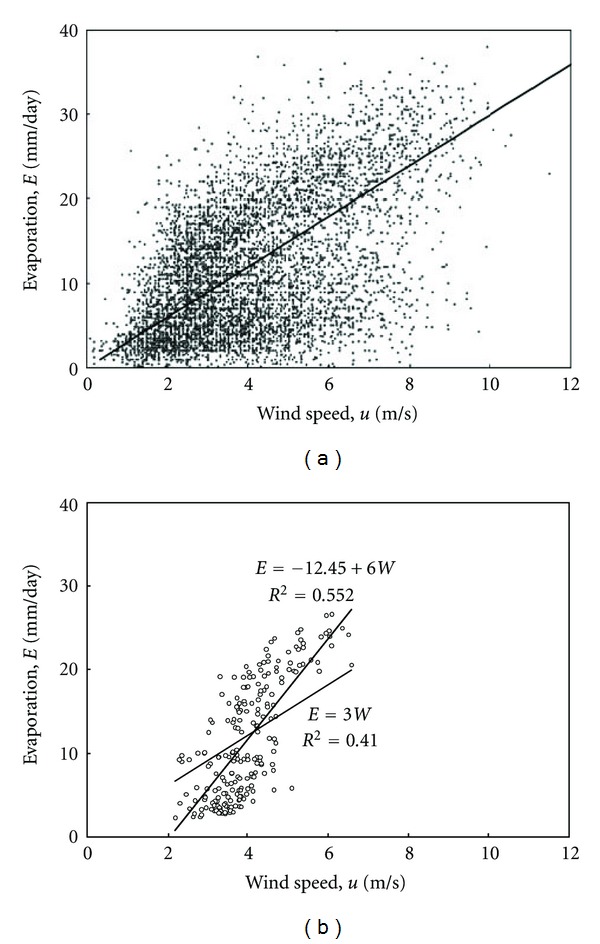
Evaporation as a function of wind speed for the data of Kuwait from 1993 to 2009. The solid curves represent suggested relations fitted using regression analysis. (a) Daily average data; (b) monthly average data.

The meteorological data of Kuwait can be examined for the suitability of fitting this type of regression model. Figures 2(a), 3(a), and 4(a) present the relation between evaporation and the other climatological parameters of temperature, relative humidity, and wind speed on a daily basis. Obviously, this time scale presentation shows high scatter in the pattern of the data by which the average individual relations expressing evaporation in terms of the other parameters become difficult to estimate accurately. In order to smooth the pattern and recognize the most suitable model form, the data can possibly be plotted on a monthly average basis. This is shown in Figures 2(b), 3(b), and 4(b) with substantial reduction in scatter compared to that of the daily average basis.

A general multiple regression model expressing the evaporation in terms of those climatological parameters can initially be assumed as

(5) $$\begin{array}{c} E_{i}=b_{1}T_{i}+b_{2}H_{i}+b_{3}u_{i}, \end{array}$$

where *E* is pan evaporation (mm day^−1^); *T* is temperature (°C); *H* is relative humidity (%); *u* is wind speed (m s^−1^). Based on the classical assumptions of multiple regression modeling, the above equation suggests linear correlations between the evaporation and the independent variables. However, Figures 2 and 3 show a definite curvilinear appearance for the relations of evaporation with temperature and relative humidity. It can be shown that those relations are best expressed correspondingly as power and exponential functions such that

(6) $$\begin{array}{c} E=bT^{\alpha }=0.04T^{1.69}, \end{array}$$

(7) $$\begin{array}{c} E=be^{\alpha H}=40.5e^{-0.038H}, \end{array}$$

and the above multiple regression equation can thus be linearized by transforming the independent variables of temperature and relative humidity as

(8) $$\begin{array}{c} T^{*}=T^{1.69}, \end{array}$$

(9) $$\begin{array}{c} H^{*}=e^{-0.038H}. \end{array}$$

Accordingly, the general regression model considering all climatological parameters examined becomes

(10) $$\begin{array}{c} E_{i}=b_{1}T_{i}^{1.69}+b_{2}e^{-0.038H_{i}}+b_{3}u_{i} \end{array}$$

and is in the linearized form

(11) $$\begin{array}{c} E_{i}=b_{1}T_{i}^{*}+b_{2}H_{i}^{*}+b_{3}u_{i}. \end{array}$$

A measure of the strength of a linear association between two variables can be presented by examining the correlation coefficients before and after the transformation.  Table 2 presents this analysis, showing improved correlations for both temperature and relative humidity after applying the transformation. This is evident for both data patterns of daily and monthly bases.

It is worth mentioning that (11) neglects the influence of other meteorological parameters on evaporation rates, as it considers an intercept equal to zero, that is, *b*_0_ = 0. Regarding the effect of wind speed, although Figure 4(b) suggests that a model with a nonzero intercept can account for a linear correlation with evaporation of the form

(12) $$\begin{array}{c} E=b_{0}+bu, \end{array}$$

regression might not result with a significant fit for the coefficients because of the considerable data scatter, regardless of the time scale chosen whether of a daily or monthly average basis. It is possible though to assume that the intercept of this correlation is equal to zero

(13) $$\begin{array}{c} E=bu. \end{array}$$

Although this assumption results with a fitting accuracy less significant for the average data presented on a monthly basis, Figure 4(a) shows that the nonzero intercept trend constitutes a possible relation fitted by eye for the data on daily basis.

## 4. Model Selection

For a multiple linear regression, model building by a variable selection procedure involves many steps reported in the literature. Typically, there are two aspects here to consider: selecting the number of variables for the models and evaluating each model selected. Including more variables in the model is not necessarily better, and it may result in overfitting. Such a model will perform poorly when applied to a new sample drawn from the same population. There is no specific test to determine the best number of variables included in the model. A possible strategy that can be applied here is to first enter the independent variables in the model one by one. Then the best of all one-variable models can be chosen as Model 1. Next all combinations of two-variable models are considered, and the best pair is chosen as Model 2. The full model with the three variables can be selected as Model 3. The three selected models can then be evaluated and discussed. The range of monthly data from January 1993 until December 2006 with 179 measurements can be used to fit all the models, and the remaining data up to December 2009 with 37 measurements may be employed to verify the performance of the final model chosen.

Suitable quantitative criteria to evaluate the accuracy of the models will be needed. A possible one is the coefficient of determination *R* ^2^, which describes the percentage of total variation explained by the model. A high *R* ^2^ value close to 1.0 indicates a good model fit with observed data. However, *R* ^2^ also increases as the number of explanatory variables used in the model increases even if these variables are not significant in explaining the variability of the dependent variable. Hence, it is preferable to use the adjusted *R*_*a*_^2^ defined as

(14) $$\begin{array}{c} R_{a}^{2}=1-\frac{n-1}{n-p}\times \left(1-R^{2}\right), \end{array}$$

where *n* is sample size; *p* is number of explanatory variables. In addition to the coefficient of determination, the performance of the models can be evaluated using statistical error tests such the Mean Absolute Percentage Error (MAPE), Root Mean Square Error (RMSE), and Nash-Sutcliffe equation (NSE). These indicators can be calculated as follows:

(15) MAPE=1n∑i=1n|yicalculated−yiobservedyiobserved×100|,RMSE=1n∑i=1n(yicalculated−yiobserved)2,NSE=1−∑i=1n(yiobserved−yicalculated)2∑i=1n(yiobserved−yi¯observed)2,

where $\bar{y}$ is the mean value of the dependent variable. For better data modeling, MAPE and RMSE statistics should be closer to zero. The NSE criterion compares the model performance to the use of the mean value of the dependent variable as an estimate. Given a perfect fit, the NSE criterion is equal to 1.0; whereas if the model is worse than the mean value of the dependent variable, the NSE statistic will be negative. In general, one can expect satisfactory results from a model with NSE criterion higher than 0.8.

Table 3 presents the accuracy measures for all possible models. In all one-variable models, the best variable based on all evaluation criteria is temperature, termed Model 1. For all combinations of two-variable models, the best two parameters based on all evaluation criteria are temperature and relative humidity, termed Model 2. Now Model 3 considers all parameters of temperature, relative humidity, and wind speed. The three models have the forms:

(16) Model  1, E=0.0422T1.69,Model  2, E=0.028T1.69+15e−0.038H,Model  3, E=0.024T1.69+18e−0.038H+0.033u.

Among the three models selected, Model 2 is considered the best one based on all the evaluation criteria shown in Table 3, while Model 1 has the lowest performance. This finding is emphasized further by recognizing that the tests of MAPE and RMSE have corresponding percentages of difference between Model 2 and Model 1 equal to 28% and 22%. 

While Model 1 has the lowest performance among the three models, it has the advantage of simplicity as it can estimate evaporation from only one climatological parameter of temperature. From a practical point of view, this model can be considered suitable to serve as a tool to estimate evaporation when input meteorological variables are insufficient. Model 3 has the advantage of including all the three parameters examined in this study; however, the accuracy of this model can be influenced by the high scatter found in the data of wind speed. Model 2 excludes wind speed by taking the advantage of the better performance of the two parameters of temperature and relative humidity. The ANOVA analysis and the regression results are shown for the three models in Tables 4 and 5, respectively. For the three models, the *F* test statistic provides a strong evidence of the presence of a satisfactorily significant linear trend between the evaporation and the other variables. However, as it was expected for Model 3, the *P* value for the wind speed variable is large, suggesting that the null hypothesis of the slope being equal to zero is true. Accordingly, Model 2 can be chosen here as the best evaporation model form.

**Figure 5:**
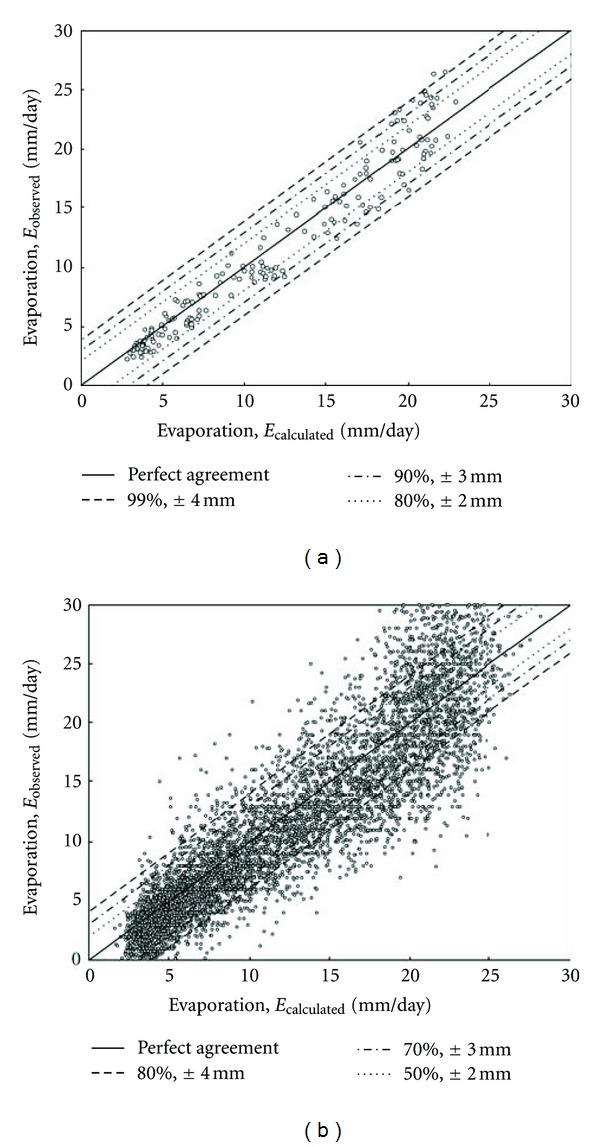
Observed versus calculated evaporation values based on Model 2 for the data of Kuwait from 1993 to 2006 (calibration range). (a) Monthly average data; and (b) daily average data.

Owing to the reason that the accuracy of results presented previously are based on monthly average data, it is crucial to test the performance of Model 2 using the daily average data. For this time scale, the accuracy results of Model 2 based on *R*_*a*_^2^, MAPE, RMSE, and NSE are equal to 0.84, 44.4, 3.4 mm, and 0.807, respectively. Although these results are satisfactory, the performance of the model for the daily data is lower than that for the monthly data. This lower performance is due to the higher scatter observed in the daily data pattern as shown in Figure 5, plotting the observed versus calculated evaporation values for both daily and monthly average data. Here, the solid line represents the condition of perfect agreement, and the other lines represent discrepancies of ±2 mm, ±3 mm, and ±4 mm. It is seen that the higher scatter in the daily evaporation data is evident. The percentage of the monthly (daily) average evaporation values calculated with a discrepancy up to ±2 mm is 80% (50%), up to ±3 mm is 90% (70%), and up to ±4 mm is 99% (80%).

**Figure 6:**
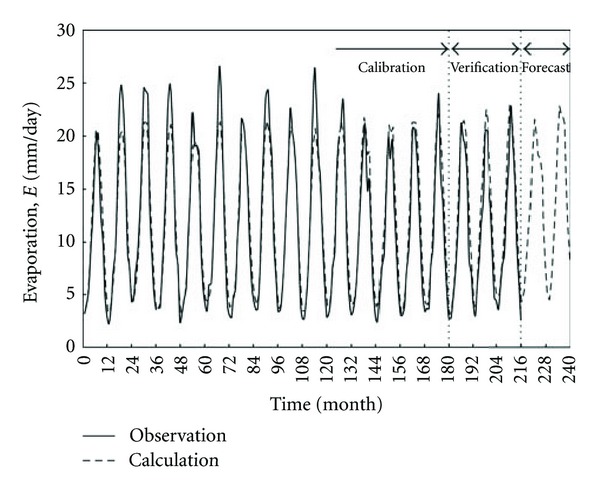
Evaporation as a function of time in months for Model 2. Calibration is performed for the duration from January 1993 (corresponding to month number 1) to December 2006 (month number 179); verification is performed for the duration from January 2007 (month 180) to December 2009 (month 216); forecast is performed for the duration from January 2010 (month 217) to December 2011 (month 240).

Model 2 can be used to provide forecasts. Initially, the model accuracy will be verified by using the range of evaporation data from January 2007 to December 2009, with 37 measurements, which has not been used for model calibration. The monthly average meteorological data can be adopted for this analysis.  Figure 6 shows a reasonable agreement between the observed and calculated evaporation values for the entire range of data from January 1993 to December 2009. The same figure also shows that the model is successful in representing most of the seasonal variation patterns during the years. Regarding the verified range, the model produces accuracy values of *R*_*a*_^2^, MAPE, RMSE, and NSE equal to 0.973, 13.36, 1.84 mm, and 0.921, respectively. These values are acceptable compared to the corresponding ones obtained for the range of model calibration shown in Table 3. It is also interesting to examine the model accuracy within this range in terms of the percentage of discrepancy criterion. Here, for the verified range, the percentage with the model discrepancy up to ±2 mm is 57%, up to ±3 mm is 82%, and up to ±4 mm is 98%.

Since the verification results are acceptable, forecasts can be produced by Model 2, given that the conditions used to derive the model remain the same. Based on the previous verification range, the mean forecasting error is assumed to be nearly within accuracy of *R*_*a*_^2^, MAPE, RMSE, and NSE equal to 0.973, 13.36, 1.84 mm, and 0.921, respectively.  Figure 6 presents two years ahead of monthly average evaporations for the period from 2010 to 2011, with 24 data points. As can be seen, the evaporation rates follow nearly the same seasonal pattern recognized for the monthly average data.

## 5. Conclusions

This study derived empirical relations for modeling pan evaporation suitable for application in Kuwait. Plotting the daily data on a monthly average basis has smoothened the high scatter in the pattern by which the relations between evaporation and the independent variables of temperature, relative humidity and wind speed became possible to estimate more accurately. The transformations considered for both variables of temperature and relative humidity by using the corresponding power and exponential functions have improved the correlation results. While these transformations are applicable locally for the meteorological data of Kuwait, it is hoped that this will bring the attention of others to examine whether such correlations exist universally in data collected from other locations. In general, the three models chosen here provided acceptable results with reasonable accuracy. Model 2 has been considered as the best one based on the evaluation criteria employed, while Model 1, although it produced the lowest performance values, has the advantage of simplicity as it requires only one climatological parameter of temperature to estimate evaporation. It is also shown that Model 2 is capable of simulating the seasonal variation pattern typically observed with monthly average evaporation data.

## Figures and Tables

**Table 1:** Descriptive statistics of daily average meteorological data for Kuwait from 1993 to 2009.

	Evaporation	Temperature	Relative humidity	Wind speed
	(mm/day)	(°C)	(%)	(m/s)
Mean	11.66	26.82	38.54	4.03
Standard deviation	7.85	9.68	20.48	1.86
Sample variance	61.64	93.65	419.37	3.44
Kurtosis	−0.38	−1.35	−0.88	−0.15
Skewness	0.68	−0.16	0.42	0.62
Range	39.90	38.04	92.00	11.36
Minimum	0.10	5.14	5.50	0.10
Maximum	40.00	43.18	97.50	11.46

**Table 2:** Correlation coefficients of daily and monthly average meteorological data for Kuwait from 1993 to 2009.

		**	Evaporation	Temperature	Humidity	Wind speed
Untransformed *T* and *H*	Monthly	Evaporation	1.00			
		Temperature	0.94	1.00		
		Humidity	−0.92	−0.92	1.00	
		Wind speed	0.74	0.59	−0.67	1.00
	Daily	Evaporation	1.00			
		Temperature	0.86	1.00		
		Humidity	−0.81	−0.78	1.00	
		Wind speed	0.53	0.32	−0.34	1.00

Transformed *T* and *H*	Monthly	Evaporation	1.00			
		Temperature	0.96	1.00		
		Humidity	0.95	0.93	1.00	
		Wind speed	0.74	0.60	0.72	1.00
	Daily	Evaporation	1.00			
		Temperature	0.87	1.00		
		Humidity	0.84	0.82	1.00	
		Wind speed	0.53	0.32	0.39	1.00

**Table 3:** Fitting accuracy of regression models using monthly average meteorological data for Kuwait from 1993 to 2006 (calibration range).

Variable	Accuracy measure			
	*R*_*a*_^2^	MAPE	RMSE (mm)	NSE
One-variable models				
*T*	0.972	16.27	2.05	0.9121
*H*	0.971	16.15	2.24	0.8945
*u*	0.843	72.65	5.30	0.4100

Two-variable models				
*T*, *H*	0.980	12.74	1.68	0.9405
*T*, *u*	0.977	17.38	2.01	0.9150
*H*, *u*	0.972	17.68	2.22	0.8968

Three-variable model				
*T*, *H*, *u*	0.979	13.23	1.69	0.9402

**Table 4:** ANOVA results for the suggested models fitted using monthly average meteorological data for Kuwait from 1993 to 2006 (calibration range).

Model number		df	SS	MS	*F*	Significance *F *
1	Regression	3	32722.44	10907.48	4718.01	0.000
	Residual	176	434.52	2.47		
	Total	179	33156.96			

2	Regression	2	32721.38	16360.69	6648.19	0.000
	Residual	177	435.58	2.46		
	Total	179	33156.96			

3	Regression	1	32375.68	32375.68	7376.18	0.000
	Residual	178	781.28	4.39		
	Total	179	33156.96			

df: degrees of freedom, SS: sum of squares, and MS: mean square.

**Table 5:** Regression results for the suggested models fitted using monthly average meteorological data for Kuwait from 1993 to 2006 (calibration range).

Model number		Coefficients	Standard error	*t* stat	*P* value
1	Intercept	0			
	Temperature	0.024	0.002	9.5010	0.000
	Humidity	18	1.875	11.450	0.000
	Wind speed	0.033	0.074	0.6560	0.513

2	Intercept	0			
	Temperature	0.028	0.002	9.9750	0.000
	Humidity	15	1.799	11.852	0.000

3	Intercept	0			
	Temperature	0.0422	0.002	85.885	0.000

## References

[1] Molina Martínez JM, Martínez Alvarez V, González-Real MM, Baille A (2006). A simulation model for predicting hourly pan evaporation from meteorological data. *Journal of Hydrology*.

[2] Shirsath PB, Singh AK (2010). A comparative study of daily pan evaporation estimation using ANN, regression and climate based models. *Water Resources Management*.

[3] Gallego-Elvira B, Baille A, Martín-Górriz B, Martínez-Álvarez V (2010). Energy balance and evaporation loss of an agricultural reservoir in a semi-arid climate (south-eastern Spain). *Hydrological Processes*.

[4] Abou El-Magd IH, Ali EM (2012). Estimation of the evaporative losses from lake Nasser, Egypt using optical satellite imagery. *International Journal of Digital Earth*.

[5] Xu CY, Singh VP (1998). Dependence of evaporation on meteorological variables at different time-scales and intercomparison of estimation methods. *Hydrological Processes*.

[6] Bouchet RJ (1963). *Evapotranspiration Reelle Evapotranspiration Potentielle, Signification Climatique*.

[7] Brutsaert W (2005). *Hydrology: An Introduction*.

[8] Vallet-Coulomb C, Legesse D, Gasse F, Travi Y, Chernet T (2001). Lake evaporation estimates in tropical Africa (Lake Ziway, Ethiopia). *Journal of Hydrology*.

[9] Gavin H, Agnew CA (2004). Modelling actual, reference and equilibrium evaporation from a temperate wet grassland. *Hydrological Processes*.

[10] Chang FJ, Chang LC, Kao HS, Wu GR (2010). Assessing the effort of meteorological variables for evaporation estimation by self-organizing map neural network. *Journal of Hydrology*.

[11] Penman HL (1948). Natural evaporation from open water, bare soil and grass. *Proceedings of the Royal Society A*.

[12] Stanhill G (1994). Changes in the rate of evaporation from the Dead Sea. *International Journal of Climatology*.

[13] Fitzpatrick EA (1963). Estimates of pan evaporation from mean maximum temperature and vapor pressure. *Journal of Applied Meteorology*.

[14] Cahoon JE, Costello TA, Ferguson JA (1991). Estimating pan evaporation using limited meteorological observations. *Agricultural and Forest Meteorology*.

[15] Crago RD, Brutsaert W (1992). A comparison of several evaporation equations. *Water Resources Research*.

[16] Rotstayn LD, Roderick ML, Farquar GD (2006). A simple pan-evaporation model for analysis of climate simulations: evaluation over Australia. *Geophysical Research Letters*.

[17] Tabari H, Marofi S, Sabziparvar AA (2010). Estimation of daily pan evaporation using artificial neural network and multivariate non-linear regression. *Irrigation Science*.

[18] Fennessey NM, Vogel RM (1996). Regional models of potential evaporation and reference evapotranspiration for the northeast USA. *Journal of Hydrology*.

[19] Linacre ET (1977). A simple formula for estimating evaporation rates in various climates, using temperature data alone. *Agricultural Meteorology*.

[20] Hargreaves GH, Samani ZA (1982). Estimating potential evapotranspiration. *Journal of the Irrigation & Drainage Division*.

[21] Abo-Ghobar HM (1993). Evaporation and drift losses from sprinkler irrigation systems under hot and dry conditions. *Journal of King Saud University*.

[22] Al-Ghobari HM (2000). Estimation of reference evapotranspiration for southern region of Saudi Arabia. *Irrigation Science*.

[23] Al-Saud MI (2009). Reduction of evaporation from water surfaces—preliminary assessment for Riyadh Region, Kingdom of Saudi Arabia. *Journal of Engineering and Applied Sciences*.

[24] Almedeij J (2012 ). Modeling rainfall variability over urban areas: a case study for Kuwait. *The Scientific World Journal*.

